# Chapare Virus, a Newly Discovered Arenavirus Isolated from a Fatal Hemorrhagic Fever Case in Bolivia

**DOI:** 10.1371/journal.ppat.1000047

**Published:** 2008-04-18

**Authors:** Simon Delgado, Bobbie R. Erickson, Roberto Agudo, Patrick J. Blair, Efrain Vallejo, César G. Albariño, Jorge Vargas, James A. Comer, Pierre E. Rollin, Thomas G. Ksiazek, James G. Olson, Stuart T. Nichol

**Affiliations:** 1 Centro de Salud de Eterazama, Cochabamba, Bolivia; 2 Special Pathogens Branch, Division of Viral and Rickettsial Disease, Centers for Disease Control and Prevention, Atlanta, Georgia, United States of America; 3 Servicio Departamental de Salud, Cochabamba, Bolivia; 4 Naval Medical Research Center Detachment, Lima, Peru; 5 Centro Nacional de Enfermedades Tropicales (CENETROP), Santa Cruz, Bolivia; University of California Irvine, United States of America

## Abstract

A small focus of hemorrhagic fever (HF) cases occurred near Cochabamba, Bolivia, in December 2003 and January 2004. Specimens were available from only one fatal case, which had a clinical course that included fever, headache, arthralgia, myalgia, and vomiting with subsequent deterioration and multiple hemorrhagic signs. A non-cytopathic virus was isolated from two of the patient serum samples, and identified as an arenavirus by IFA staining with a rabbit polyvalent antiserum raised against South American arenaviruses known to be associated with HF (Guanarito, Machupo, and Sabiá). RT-PCR analysis and subsequent analysis of the complete virus S and L RNA segment sequences identified the virus as a member of the New World Clade B arenaviruses, which includes all the pathogenic South American arenaviruses. The virus was shown to be most closely related to Sabiá virus, but with 26% and 30% nucleotide difference in the S and L segments, and 26%, 28%, 15% and 22% amino acid differences for the L, Z, N, and GP proteins, respectively, indicating the virus represents a newly discovered arenavirus, for which we propose the name Chapare virus. In conclusion, two different arenaviruses, Machupo and Chapare, can be associated with severe HF cases in Bolivia.

## Author Summary

Four rodent-borne arenaviruses are known to cause hemorrhagic fever (HF) in the New World. These include Junín, Machupo, Guanarito, and Sabiá viruses, which are found in rural areas of Argentina, Bolivia, Venezuela, and Brazil, respectively. In December 2003 and January 2004, a small number of HF cases were reported in rural Bolivia in an area outside the known Machupo HF endemic zone, and sera from one fatal case was available for laboratory testing. The man had symptoms similar to those seen with other arenaviral HF cases—acute febrile illness beginning with headache, joint and muscle pain, and vomiting—and rapidly progressed to shock, bleeding, and death at 14 days post onset of illness. Virus was isolated from two of the patient's serum samples and identified as an arenavirus by reaction of virus infected cells with arenavirus-specific antibodies and by genetic detection techniques (PCR). Subsequent complete genome analysis of the virus showed the virus to be a distinct newly discovered member of the arenavirus family, and the name Chapare virus was proposed. The virus is phylogenetically related to other arenaviruses that naturally cause hemorrhagic fever in South America, particularly Sabiá virus. Physicians should consider Chapare virus as a potential etiologic agent when encountering HF cases in the region.

## Introduction

**Figure 1:**
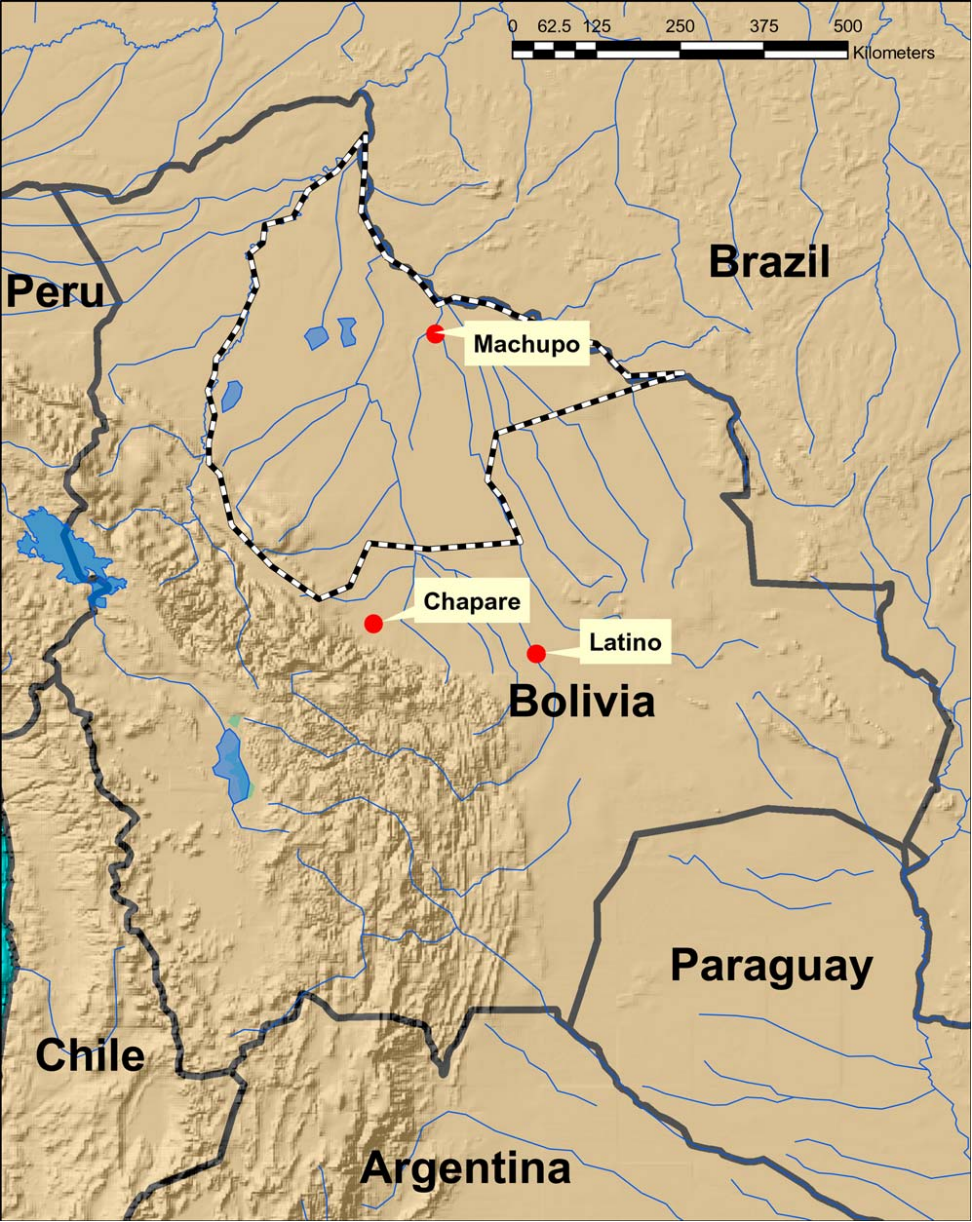
Map of Bolivia showing location of the Chapare virus-associated HF case relative to the Beni region where Machupo virus-associated HF cases originate. The Beni Department boundary is depicted by the checkered line. Multiple Machupo isolates have been recorded from the Beni Department. The single Latino and Chapare virus locations are labeled and represented as dots.

The family *Arenaviridae* is composed of largely rodent-borne viruses which are divided into Old World and New World complexes [1],[2]. Lassa and lymphocytic choriomeningitis (LCM) viruses are considered the most important of Old World arenaviruses due to their association with severe disease. The New World complex is divided into 3 major Clades (A, B and C), with Clade B containing all the hemorrhagic fever (HF) associated viruses [3],[4],[5],[6]. These are Junín, Machupo, Guanarito and Sabiá viruses, the cause of Argentine, Bolivian, Venezuelan, and Brazilian HF, respectively [1]. Three of these viruses, Junín, Machupo, and Guanarito, can be associated with large HF outbreaks and untreated case fatalities can be in excess of 30%. The clinical picture is similar for each of these diseases. Onset of symptoms follows an incubation period of 1–2 weeks. Initial symptoms often include fever, malaise, myalgia and anorexia, followed approx. 3–4 days later by headache, back pain, dizziness, nausea, vomiting, and severe prostration. Hemorrhagic and neurologic symptoms, including petechiae and bleeding gums, tremors, and lethargy are common. About a third of untreated cases go on to develop more severe neurologic and/or hemorrhagic symptoms, with diffuse echymoses, and bleeding from mucous membranes or puncture sites, and/or delirium, coma and convulsions. Machupo virus, vectored by *Calomys callosus* rodents [7], is the only known pathogenic arenavirus found in Bolivia, although another arenavirus, Latino virus, has also been isolated from *Calomys callosus* in Bolivia [8]. Despite broad distribution of this rodent host, which is thought to include the lowlands of Bolivia, east-central Brazil, Paraguay and northern Argentina [9], Machupo virus-associated HF cases have originated only in the Beni department in northeastern Bolivia (Figure 1). We report here the investigation of a fatal HF case which occurred near Cochabamba, Cochabamba Department, Bolivia in December, 2003, and identify the associated arenavirus as a unique newly discovered virus, Chapare virus.

## Results/Discussion

In late 2003 reports were received of a small cluster of HF cases in a rural area near the Chapare River, close to Cochabamba, Bolivia in the eastern foothills of the Andes (Figure 1). Exact details of the number of cases and verification of symptoms were difficult to obtain. However, a clinical description and blood specimens were available for one fatal case. This patient, a 22 year old male, had lived for the last 4 years in Samuzabeti, a small town located 35 km northeast of Villa Tunari. He was a tailor and also a farmer. Coca is the main crop in this area. He had no history of travel and no contact with any case with compatible illness for at least 4 weeks prior to his disease onset on January 3^rd^, 2004. In addition, no members of case household or other close contacts were affected. His clinical course included fever, headache, arthralgia, myalgia and vomiting with subsequent deterioration and multiple hemorrhagic signs and death on January 17^th^, 2004 (14 days post onset). Based on these symptoms, the patient was initially suspected of having yellow fever or dengue HF. However, initial tests for these agents were negative. Initial IgM, IgG, antigen capture and RT-PCR testing for Machupo virus or related arenaviruses were also negative.

Patient specimens were sent to the biosafety level 4 (BSL4) containment laboratory at the Special Pathogens Branch in Atlanta where virus isolation attempts could be performed. These specimens consisted of 4 acute phase sera, collected on days 4, 7, 9 and 14 post onset of disease. Both the day 7 and day 9 sera yielded a non-cytopathic virus by growth in Vero E6 cells. These were identified by immunofluorescent antibody (IFA) staining with rabbit polyvalent hyperimmune serum raised against South American arenaviruses previously known to be associated with HF (Guanarito, Machupo, and Sabiá). RT-PCR analysis of the virus isolate RNAs amplified a 481 bp fragment which yielded nucleotide sequence related to known New World Clade B arenaviruses (which includes all the South American HF associated arenaviruses). Full length virus genome sequences were successfully determined for the virus isolated from the day 9 post onset bleed (designated strain 810419) by RT-PCR and sequence analysis followed by primer walking utilizing newly derived sequence information. The full length S segment was amplified by using the 19C primer designed based on the conserved RNA termini of New World Arenaviruses [10], whereas the L segment was amplified in multiple sections using a variety of primers (sequences available on request). Sequence analysis of the complete S and L segments confirmed that this virus, proposed name Chapare, was a unique member of the Clade B New World arenaviruses [3],[4],[5],[10],[11]. The virus was found to be most closely related to Sabiá virus, but with 26 and 30% nucleotide difference in the complete S and L segments, and 26, 28, 15 and 22% amino acid differences for the L, Z, N and GP proteins, respectively (Tables 1 and S1). The genetic differences between Chapare virus and other Clade B viruses range from 36–40% for the complete S segment and 39–40% for the complete L segment (data not shown). These nucleotide and amino acid sequence divergence levels are in excess of those seen among strains of the same species of New World arenavirus (Tables 2 and S1) [12],[13],[14]. For instance, the greatest difference seen between complete S segments of virus strains is 14% (within Allpahuayo virus strains) and 10% for the complete L segment (among Machupo virus strains) [15].

**Table 1:** Differences between Chapare virus and its closest relative, Sabiá, are similar to differences between other distinct species of arenavirus

Virus	Nucleotide^a^		Amino Acid^b^			
	S segment	L segment	GPC	NP	L	Z
Chapare to Sabiá	26	30	22	15	26	28
Machupo to Junín	25–27	31	25–27	11–14	25	18–20
Machupo to Tacaribe	31–32	33	32–33	19–20	27–28	21
Amapari to Guanarito	27	32	29	14	28	28–32
Paraná to Flexal	29	n/a^c^	17	21	n/a	n/a

a Complete nucleotide segments only

b Complete amino acid sequences only

c Complete segment or gene sequence is not available for one or both viruses

**Table 2:** Differences among strains of the same species of arenavirus

Virus	Nucleotide^a^		Amino Acid^b^			
	S segment	L segment	GPC	NP	L	Z
Allpahuayo	**14**	n/a^c^	2	2	n/a	n/a
Bear Canyon	3	3	2	1	2	0
Catarina	9	n/a	5	2	n/a	n/a
Flexal	0.1	n/a	0	0	n/a	n/a
Guanarito	2	n/a	1	0	n/a	11
Junín	7	3	2	4	2	1
Machupo	13	**10**	5	3	5	6
Pichindé	11	n/a	5	3	n/a	n/a
Whitewater Arroyo	0.4	n/a	0	1	n/a	n/a

a Complete nucleotide segments only

b Complete amino acid sequences only

c Complete segment or gene sequence is not available for more than one strain

**Figure 2:**
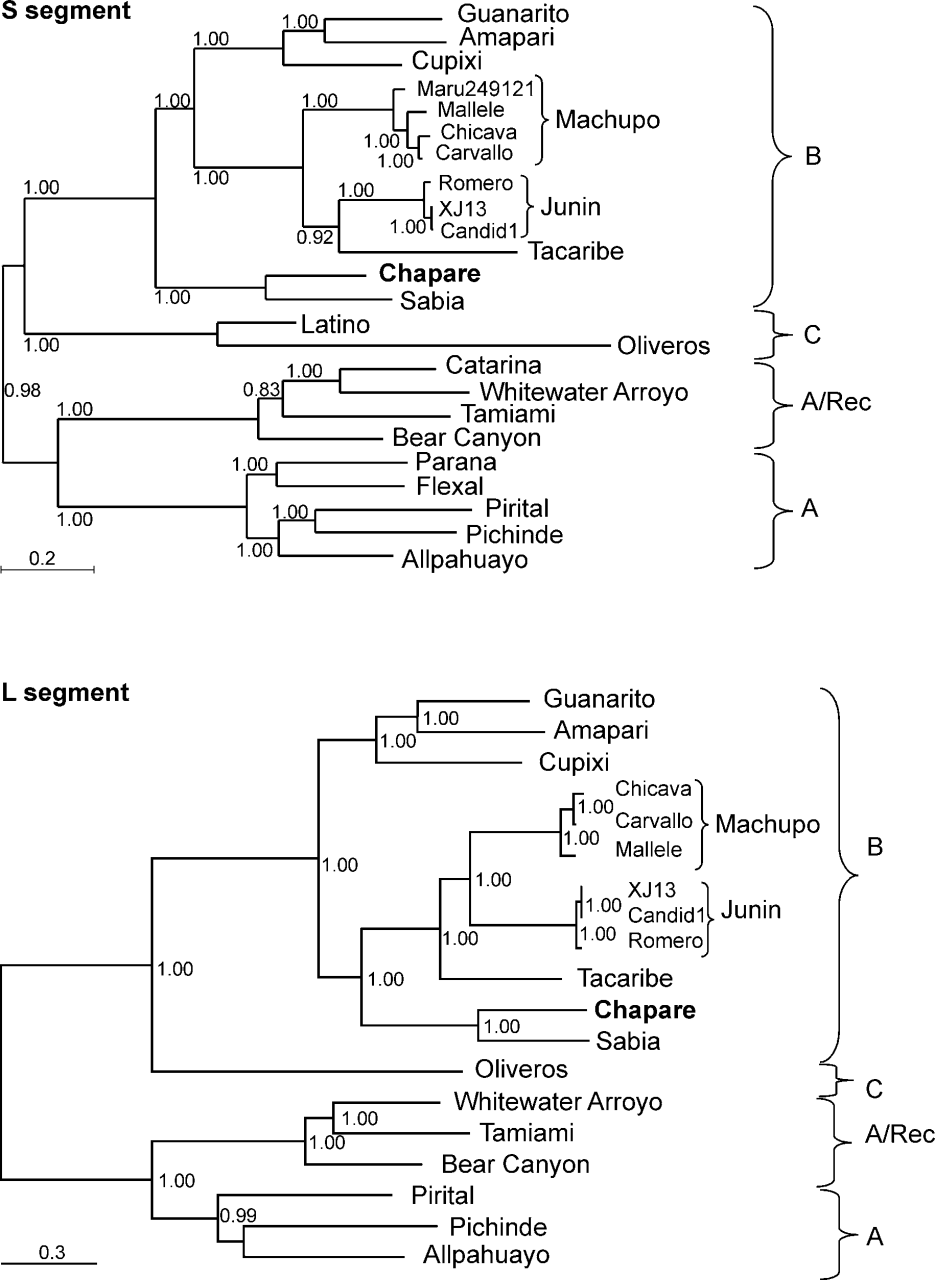
Phylogenetic analysis of the complete S and L RNA segments of New World arenaviruses. Complete S and L segments for New World arenaviruses were analyzed by Bayesian inference of phylogeny (MrBayes3.1.2) using the sequence of Pichindé virus as the outgroup. Multiple strains are grouped with small brackets and large brackets group the arenavirus Clades: A, A/Rec, B, and C. The Genbank accession numbers for the S segment analysis include: Allpahuayo (AY012686), Amapari (AF485256), Bear Canyon (AY924392), Catarina (DQ865245), Chapare (EU260463), Cupixi (AF512832), Flexal (AF485257), Guanarito (NC_005077), Junín (AY619641, NC_005081, AY746353), Latino (AF512830), Machupo (AY924208, AY619645, AY924202, NC_005078), Oliveros (U34248), Paraná (AF485261), Pichindé (NC_006447), Pirital (NC_005894), Sabiá (NC_006317), Tacaribe (NC_004293), Tamiami (AF485263), and Whitewater Arroyo (AF485264). The Genbank accession numbers used for the L segment analysis include: Allpahuayo (NC_010249), Amapari (AY924389), Bear Canyon (AY924390), Chapare (EU260464), Cupixi (NC_010252), Guanarito (NC_005082), Junín (NC_005080, AY819707, AY619640), Machupo (AY624354, NC_005079, AY619644), Oliveros (NC_010250), Pichindé (NC_006439), Pirital (NC_005897), Sabiá (NC_006313), Tacaribe (NC_004292), Tamiami (AY924393), and Whitewater Arroyo (AY924395).

Chapare virus was found to be monophyletic with Sabiá virus on phylogenetic analysis of the nucleotide or encoded amino acid sequences of the complete S or L segment (Figure 2), or NP, GP, L or Z ORFs (data not shown). No evidence of reassortment or recombination between Chapare virus and other arenaviruses was found. There is no overall change in the structure of the trees except for the previously described [4],[5] switch of the Clade A/Rec viruses from Clade A for the NP gene to Clade B for the GP gene (data not shown).

The pathogenicity of the New World Clade B viruses correlates with the efficient interaction of their GP1 surface glycoproteins with the human cellular receptor, transferring receptor 1 (TfR1) [16],[17]. Presumably, Chapare virus will be found to have similar TfR1 binding properties, but this remains to be confirmed. Even assuming this to be true, the diversity of the GP1 amino acid sequences of Junín, Machupo, Guanarito, Sabiá and Chapare viruses is such that one cannot easily discern the GP1 domain involved in high efficiency binding to TfR1 solely on the basis of amino acid sequence alignments.

The relationship of Chapare virus from Bolivia to Sabiá virus from Brazil is intriguing. Both these virus clearly cause HF similar to that seen with Junín, Machupo and Guanarito viruses. The single HF case associated with a naturally acquired Sabiá virus infection was reported in the community of Sabiá, in Sao Paulo, Brazil in 1990 [18]. The exact site of exposure was unclear, as was the rodent reservoir. Yellow fever was the initial suspicion in the Sabiá case and that associated with the Chapare virus infection as both had associated extensive liver necrosis. More extensive liver involvement may be a feature shared between these viruses, as it is not commonly observed with HF associated with the other New World arenaviruses (although it is occasionally seen).

Due to the difficulties of working in this resource poor rural region, initial follow up efforts in the Chapare area, did not yield a more precise description of the reported cluster of cases with similar illness, and a limited ecological study did not reveal the rodent reservoir of this virus. It is hoped that more extensive studies in the area will reveal the extent to which Chapare virus poses a public health problem in this area, and shed light on the source of human infection. In summary, three arenaviruses are now known to be present in Bolivia, namely Machupo and Latino viruses (both hosted by *Calomys callosus*) and Chapare viruses (reservoir unknown). Furthermore, both Machupo and Chapare viruses are agents of fatal hemorrhagic fever in Bolivia.

## Materials and Methods

### Diagnostic Amplification & Identification

Initial virus genetic detection and analysis was conducted on total RNA extracted from infected Vero E6 cells, using Tripure Isolation Reagent (Roche Applied Science, Indianapolis, IN) in a ratio of 1∶5 and incubated at room temperature for a minimum of 10 min. Total RNA was isolated by using the RNaid Kit following the manufacturer's recommendations (Qbiogene Inc., Carlsbad, CA), and the extracted RNA was reconstituted in 50 µL H_2_O. Broadly reactive Arenavirus primers used for initial identification were designed for the L polymerase gene on the L segment (L4160F, GCA GAR TTY AAA TCI AGA TT; L4393R, CCR TYI ASC CAR TCT ITI ACA TC; L4292F, GAT CAT TCI RTY GCI AAT GG; L4841R, CAI AII CCT ATA AAI CCW GAT G) [19] and the glycoprotein gene on the S segment (GP878+, GAC RTG CCW GGI GGI TAY TG; GP1126-, TAC CAA AAT TTG TGT ART TRC ART AIG G; GP1153+, CCT TAY TGY AAY TAC ACI AAA TTT TGG T; GP1396-, ATG TGY CTR TGI GTI GGI AW).

Reverse Transcription (RT) was done using 2.5 µL of RNA in a 25 µL total reaction volume and AMV RT (Promega Biosciences, San Luis Obispo, CA) at 42°C for 1 hr. Subsequent PCR amplification using FastStart Taq DNA Polymerase with GC-rich solution (Roche) was performed using 5 µL of the completed RT reaction in a 25 µL reaction volume with the following cycling conditions: 2 min at 95°C, (36 cycles of 1 min at 95°C, 1 min at 45°C, 2 min at 72°C), and a final elongation of 10 min at 72°C. Resulting DNA products were visualized and purified using a 1% agarose gel, and the Qiagen Gel Extraction Kit (Qiagen, Valencia, CA). PCR products were sequenced directly (without cloning) using the corresponding primers in a BigDye Terminator v3.1 reaction on a 3100 Genetic Analyzer (Applied Biosystems, Foster City, CA). Sequence was further analyzed using Sequencher (Gene Codes Corporation, Ann Arbor, MI).

### Complete Genome Amplification & Analysis

To obtain full length sequence for each segment, alignments of all New World arenavirus complete genomes were used to design primers for the conserved regions (available upon request). The full length S segment was generated following the Thermoscript RT-PCR system's directions (Invitrogen, Carlsbad, CA) and using the 19C primer [10]. Reverse transcription was conducted at 55°C, while the PCR profile was the same as stated above with an increased extension time of 4 minutes.

Different fractions of the full-length L RNA were amplified using 2-step or 1-step RT-PCR protocols and following the manufacturer recommendations. Briefly, cDNA was synthesized in the first approach using 10 µl of purified RNA, specific primers, dNTPs and Superscript III (Invitrogen) in 20 µl reactions. Amplification reactions were done using 5 µl of cDNA, specific primers, dNTPs and Platinum Taq DNA polymerase High Fidelity (Invitrogen) in 50 ul reactions. Alternatively, 1-step RT-PCR were performed using 5 µl of RNA, dNTPs and the enzyme blend provided by the SuperScript III One-Step RT-PCR System with Platinum Taq High Fidelity (Invitrogen) in a 50 µl reactions. Amplification reactions were analyzed in TBE/agarose gels and DNA bands purified using QIAquick Gel Extraction Kit (Qiagen). Sequencing reactions were done as described above.

### Phylogenetic Analysis

All full length S and L segment sequences available in Genbank were used to compute pairwise uncorrected genetic distances using PAUP 4.0b10 (Sinauer Associates) for the following viruses: Allpahuayo, Amapari, Chapare, Flexal, Guanarito, Junín, Machupo, Paraná, Pichindé, Pirital, Sabiá, Tacaribe, Tamiami, and Whitewater Arroyo.

A representative sub-set of full length sequences (omitting multiple near identical variants of the same virus) were included in a Bayesian phylogenetic analysis. Sequence alignments were done with ClustalX [20] with manual adjustments and phylogenetic analysis was done with MrBayes3.1.2 [21] using the GTR+I+G model in 2 runs of 500,000 generations using the sequence of Pichindé virus as the outgroup.

## Supporting Information

Table S1 Amino acid distances for complete L and Z genes and nucleocapsid and glycoprotein genes of the New World arenaviruses
(0.34 MB PDF)
