# Supplementary material for: Chapare Virus, a Newly Discovered Arenavirus Isolated from a Fatal Hemorrhagic Fever Case in Bolivia
Source: PLoS Pathog. 2008 Apr 18;4(4):e1000047. doi: 10.1371/journal.ppat.1000047 (PMC2277458; doi:10.1371/journal.ppat.1000047)
Supplement: Table S1 — Amino acid distances for complete L and Z genes and nucleocapsid and glycoprotein genes of the New World arenaviruses (0.34 MB PDF) [file ppat.1000047.s001.pdf]

Amino acid distances for complete L and Z genes of the new world arenaviruses*

		1	2	3	4	5	6	7	8	9	10	11	12	13	14	15	16	17	18	19	20	21	22	23	24	25	26	27	28	29	30	31	32	33	34	35	36	37	38	39	40	41	42	43	44	45
1	ALL_CLHP2472	—	56	43	44	55	56	n/a‡	n/a	n/a	n/a	n/a	n/a	n/a	n/a	n/a	n/a	n/a	56	56	56	56	56	56	n/a	55	55	n/a	n/a	n/a	n/a	n/a	n/a	n/a	56	54	36	n/a	n/a	n/a	n/a	34	56	55	42	43
2	AMA_BeAn70563	54	—	56	56	39	31	n/a	n/a	n/a	n/a	n/a	n/a	n/a	n/a	n/a	n/a	n/a	28	39	39	39	39	39	n/a	38	38	n/a	n/a	n/a	n/a	n/a	n/a	n/a	38	49	55	n/a	n/a	n/a	n/a	55	39	40	56	56
3	BCN_A0060209	31	54	—	2	56	55	n/a	n/a	n/a	n/a	n/a	n/a	n/a	n/a	n/a	n/a	n/a	55	56	56	56	56	56	n/a	56	55	n/a	n/a	n/a	n/a	n/a	n/a	n/a	56	55	45	n/a	n/a	n/a	n/a	43	56	57	28	27
4	BCN_AVA0070039	31	54	0	—	56	55	n/a	n/a	n/a	n/a	n/a	n/a	n/a	n/a	n/a	n/a	n/a	56	56	56	56	56	56	n/a	56	55	n/a	n/a	n/a	n/a	n/a	n/a	n/a	55	54	46	n/a	n/a	n/a	n/a	43	55	57	28	27
5	CHA_810419	59	41	55	55	—	41	n/a	n/a	n/a	n/a	n/a	n/a	n/a	n/a	n/a	n/a	n/a	40	38	38	38	38	38	n/a	37	38	n/a	n/a	n/a	n/a	n/a	n/a	n/a	38	49	55	n/a	n/a	n/a	n/a	55	26	38	56	56
6	CPX_BeAn119303	58	29	56	56	39	—	n/a	n/a	n/a	n/a	n/a	n/a	n/a	n/a	n/a	n/a	n/a	31	40	40	40	40	40	n/a	39	39	n/a	n/a	n/a	n/a	n/a	n/a	n/a	39	50	56	n/a	n/a	n/a	n/a	56	41	40	56	55
7	GTO_VHF1608	55	32	52	52	38	28	—	n/a	n/a	n/a	n/a	n/a	n/a	n/a	n/a	n/a	n/a	n/a	n/a	n/a	n/a	n/a	n/a	n/a	n/a	n/a	n/a	n/a	n/a	n/a	n/a	n/a	n/a	n/a	n/a	n/a	n/a	n/a	n/a	n/a	n/a	n/a	n/a	n/a	n/a
8	GTO_AV97021119	55	28	52	52	35	26	9	—	n/a	n/a	n/a	n/a	n/a	n/a	n/a	n/a	n/a	n/a	n/a	n/a	n/a	n/a	n/a	n/a	n/a	n/a	n/a	n/a	n/a	n/a	n/a	n/a	n/a	n/a	n/a	n/a	n/a	n/a	n/a	n/a	n/a	n/a	n/a	n/a	n/a
9	GTO_VHF1750	56	32	53	53	39	26	6	7	—	n/a	n/a	n/a	n/a	n/a	n/a	n/a	n/a	n/a	n/a	n/a	n/a	n/a	n/a	n/a	n/a	n/a	n/a	n/a	n/a	n/a	n/a	n/a	n/a	n/a	n/a	n/a	n/a	n/a	n/a	n/a	n/a	n/a	n/a	n/a	n/a
10	GTO_VHF3990	54	31	52	52	36	29	11	8	9	—	n/a	n/a	n/a	n/a	n/a	n/a	n/a	n/a	n/a	n/a	n/a	n/a	n/a	n/a	n/a	n/a	n/a	n/a	n/a	n/a	n/a	n/a	n/a	n/a	n/a	n/a	n/a	n/a	n/a	n/a	n/a	n/a	n/a	n/a	n/a
11	GTO_S56764	55	28	52	52	35	26	8	1	6	7	—	n/a	n/a	n/a	n/a	n/a	n/a	n/a	n/a	n/a	n/a	n/a	n/a	n/a	n/a	n/a	n/a	n/a	n/a	n/a	n/a	n/a	n/a	n/a	n/a	n/a	n/a	n/a	n/a	n/a	n/a	n/a	n/a	n/a	n/a
12	GTO_CVH961104	55	29	52	52	36	27	6	4	5	5	2	—	n/a	n/a	n/a	n/a	n/a	n/a	n/a	n/a	n/a	n/a	n/a	n/a	n/a	n/a	n/a	n/a	n/a	n/a	n/a	n/a	n/a	n/a	n/a	n/a	n/a	n/a	n/a	n/a	n/a	n/a	n/a	n/a	n/a
13	GTO_CVH950801	55	31	52	52	36	28	7	5	6	6	4	1	—	n/a	n/a	n/a	n/a	n/a	n/a	n/a	n/a	n/a	n/a	n/a	n/a	n/a	n/a	n/a	n/a	n/a	n/a	n/a	n/a	n/a	n/a	n/a	n/a	n/a	n/a	n/a	n/a	n/a	n/a	n/a	n/a
14	GTO_CVH960102	55	31	52	52	36	28	7	5	6	6	4	1	0	—	n/a	n/a	n/a	n/a	n/a	n/a	n/a	n/a	n/a	n/a	n/a	n/a	n/a	n/a	n/a	n/a	n/a	n/a	n/a	n/a	n/a	n/a	n/a	n/a	n/a	n/a	n/a	n/a	n/a	n/a	n/a
15	GTO_CVH960103	55	32	52	52	38	28	7	6	6	7	5	2	1	1	—	n/a	n/a	n/a	n/a	n/a	n/a	n/a	n/a	n/a	n/a	n/a	n/a	n/a	n/a	n/a	n/a	n/a	n/a	n/a	n/a	n/a	n/a	n/a	n/a	n/a	n/a	n/a	n/a	n/a	n/a
16	GTO_CVH960302	55	32	52	52	38	28	7	6	6	7	5	2	1	1	0	—	n/a	n/a	n/a	n/a	n/a	n/a	n/a	n/a	n/a	n/a	n/a	n/a	n/a	n/a	n/a	n/a	n/a	n/a	n/a	n/a	n/a	n/a	n/a	n/a	n/a	n/a	n/a	n/a	n/a
17	GTO_CVH960101	55	31	52	52	36	28	7	5	6	6	4	1	0	0	1	1	—	n/a	n/a	n/a	n/a	n/a	n/a	n/a	n/a	n/a	n/a	n/a	n/a	n/a	n/a	n/a	n/a	n/a	n/a	n/a	n/a	n/a	n/a	n/a	n/a	n/a	n/a	n/a	n/a
18	GTO_INH95551	55	31	52	52	38	29	7	6	6	7	5	2	1	1	2	2	1	—	39	39	39	39	39	n/a	38	38	n/a	n/a	n/a	n/a	n/a	n/a	n/a	38	50	56	n/a	n/a	n/a	n/a	55	39	40	55	55
19	JUN_MC2	56	33	58	58	40	28	32	29	31	33	29	31	31	31	31	31	31	29	—	0	2	2	2	n/a	25	25	n/a	n/a	n/a	n/a	n/a	n/a	n/a	25	50	56	n/a	n/a	n/a	n/a	54	38	29	56	56
20	JUN_Rumero	56	33	58	58	40	28	32	29	31	33	29	31	31	31	31	31	31	29	0	—	2	2	2	n/a	25	25	n/a	n/a	n/a	n/a	n/a	n/a	n/a	25	50	56	n/a	n/a	n/a	n/a	54	38	29	56	56
21	JUN_Candid1	56	33	58	58	40	29	33	31	32	34	31	32	32	32	32	32	32	31	1	1	—	0	0	n/a	25	25	n/a	n/a	n/a	n/a	n/a	n/a	n/a	25	50	56	n/a	n/a	n/a	n/a	54	39	29	56	56
22	JUN_XJ44	56	33	58	58	40	29	33	31	32	34	31	32	32	32	32	32	32	31	1	1	0	—	0	n/a	25	25	n/a	n/a	n/a	n/a	n/a	n/a	n/a	25	50	56	n/a	n/a	n/a	n/a	54	39	29	56	56
23	JUN_XJ13	56	33	58	58	40	29	33	31	32	34	31	32	32	32	32	32	32	31	1	1	0	0	—	n/a	25	25	n/a	n/a	n/a	n/a	n/a	n/a	n/a	25	50	56	n/a	n/a	n/a	n/a	54	38	28	56	56
24	LAT_MARU10924	56	49	56	56	46	48	48	49	49	48	49	48	48	48	48	48	48	48	49	49	49	49	49	—	n/a	n/a	n/a	n/a	n/a	n/a	n/a	n/a	n/a	n/a	n/a	n/a	n/a	n/a	n/a	n/a	n/a	n/a	n/a	n/a	n/a
25	MAC_Mallele	54	31	56	56	36	29	34	31	33	32	31	32	32	32	32	32	32	33	20	20	20	20	20	49	—	5	n/a	n/a	n/a	n/a	n/a	n/a	n/a	4	49	56	n/a	n/a	n/a	n/a	55	37	28	55	56
26	MAC_Chicava	56	32	58	58	38	29	33	29	32	33	29	31	31	31	31	31	31	32	19	19	19	19	19	49	4	—	n/a	n/a	n/a	n/a	n/a	n/a	n/a	2	49	56	n/a	n/a	n/a	n/a	54	37	27	55	56
27	MAC_9430084	55	32	56	56	38	29	32	29	32	32	29	29	29	29	29	29	29	31	18	18	18	18	18	51	6	6	—	n/a	n/a	n/a	n/a	n/a	n/a	n/a	n/a	n/a	n/a	n/a	n/a	n/a	n/a	n/a	n/a	n/a	n/a
28	MAC_200002427	55	31	56	56	36	29	33	29	32	33	29	31	31	31	31	31	31	32	19	19	19	19	19	49	4	4	2	—	n/a	n/a	n/a	n/a	n/a	n/a	n/a	n/a	n/a	n/a	n/a	n/a	n/a	n/a	n/a	n/a	n/a
29	MAC_9301012	56	32	58	58	38	29	33	29	32	33	29	31	31	31	31	31	31	32	19	19	19	19	19	49	4	0	6	4	—	n/a	n/a	n/a	n/a	n/a	n/a	n/a	n/a	n/a	n/a	n/a	n/a	n/a	n/a	n/a	n/a
30	MAC_MARU216606	55	31	56	56	38	29	33	29	32	33	29	31	31	31	31	31	31	32	19	19	19	19	19	49	2	1	5	2	1	—	n/a	n/a	n/a	n/a	n/a	n/a	n/a	n/a	n/a	n/a	n/a	n/a	n/a	n/a	n/a
31	MAC_MARU222688	55	31	56	56	36	29	33	29	32	33	29	31	31	31	31	31	31	32	19	19	19	19	19	49	4	4	2	0	4	2	—	n/a	n/a	n/a	n/a	n/a	n/a	n/a	n/a	n/a	n/a	n/a	n/a	n/a	n/a
32	MAC_MARU249121	55	31	56	56	36	29	33	29	32	33	29	31	31	31	31	31	31	32	19	19	19	19	19	49	4	4	2	0	4	2	0	—	n/a	n/a	n/a	n/a	n/a	n/a	n/a	n/a	n/a	n/a	n/a	n/a	n/a
33	MAC_9530537	55	31	56	56	38	29	33	29	32	33	29	31	31	31	31	31	31	32	19	19	19	19	19	51	4	4	5	2	4	2	2	2	—	n/a	n/a	n/a	n/a	n/a	n/a	n/a	n/a	n/a	n/a	n/a	n/a
34	MAC_Carvallo	55	31	56	56	38	29	33	29	32	33	29	31	31	31	31	31	31	32	19	19	19	19	19	49	2	1	5	2	1	0	2	2	2	—	49	56	n/a	n/a	n/a	n/a	55	37	27	55	56
35	OLI_3229	52	45	53	53	46	46	51	46	51	47	46	48	48	48	49	49	48	48	48	48	48	48	48	22	47	46	48	47	46	46	47	47	48	46	—	54	n/a	n/a	n/a	n/a	53	50	50	55	55
36	PIC_AN3739	20	58	35	35	59	60	54	55	56	54	55	54	54	54	54	54	54	54	60	60	60	60	60	55	58	59	56	58	59	58	58	58	58	58	53	—	n/a	n/a	n/a	n/a	38	56	57	45	45
37	PIR_1743	16	53	32	32	59	55	53	53	54	53	53	53	53	53	53	53	53	53	56	56	56	56	56	59	54	55	54	54	55	54	54	54	54	54	54	26	—	n/a	n/a	n/a	n/a	n/a	n/a	n/a	n/a
38	PIR_3945	22	53	32	32	58	54	53	53	54	53	53	53	53	53	53	53	53	53	56	56	56	56	56	58	53	54	53	53	54	53	53	53	53	53	53	26	8	—	n/a	n/a	n/a	n/a	n/a	n/a	n/a
39	PIR_1645	16	54	33	33	59	54	54	54	53	54	54	54	54	54	54	54	54	54	56	56	56	56	56	60	54	55	54	54	55	54	54	54	54	54	55	24	5	9	—	n/a	n/a	n/a	n/a	n/a	n/a
40	PIR_AV97021016	21	54	32	32	58	54	54	54	53	54	54	54	54	54	54	54	54	54	56	56	56	56	56	59	53	54	53	53	54	53	53	53	53	53	55	25	11	6	6	—	n/a	n/a	n/a	n/a	n/a
41	PIR_VAV488	18	54	32	32	58	54	54	54	53	54	54	54	54	54	54	54	54	54	56	56	56	56	56	59	54	55	54	54	55	54	54	54	54	54	53	22	7	8	2	5	—	56	55	44	44
42	SAB_SPH114202	58	47	53	53	28	44	39	36	38	39	36	36	36	36	36	36	36	38	45	45	45	45	45	51	39	39	40	39	39	39	39	39	39	39	49	58	55	53	54	53	53	—	38	55	56
43	TAC	58	33	58	58	40	29	28	28	31	28	27	27	27	27	28	28	27	27	25	25	25	25	25	48	21	21	21	21	21	21	21	21	21	21	47	59	54	52	55	54	55	42	—	55	57
44	TAM_W10777	29	54	20	20	59	55	49	49	51	51	49	49	51	51	51	51	51	51	56	56	56	56	56	55	58	58	56	56	58	56	56	56	56	56	52	36	38	40	38	39	36	55	59	—	27
45	WWA_AV9310135	27	53	13	13	54	59	52	52	53	52	52	52	52	52	52	52	52	52	59	59	59	59	59	58	58	59	58	58	59	58	58	58	58	58	54	33	28	32	31	32	29	52	59	18	—

*Upper-right and lower-left of diagonal corresponds to uncorrected pairwise p distances for the complete L and complete Z amino acid sequences, respectively

‡Complete amino acid sequence is not available

Amino acid distances for complete nucleocapsid and glycoprotein genes of the new world arenaviruses*

		1	2	3	4	5	6	7	8	9	10	11	12	13	14	15	16	17	18	19	20	21	22	23	24	25	26	27	28	29	30	31	32	33	34	35	36	37	38	39	40	41	42
1	ALL_Peru	—	2	42	35	35	35	41	42	38	38	26	26	42	42	42	42	43	42	42	n/a‡	n/a	41	40	41	41	40	41	41	41	41	41	41	41	28	23	23	30	43	40	42	37	36
2	ALL_CLHP2098	2	—	30	35	35	35	41	42	38	38	25	25	42	42	42	42	43	42	42	n/a	n/a	41	40	41	41	40	41	41	41	41	41	41	41	28	23	23	30	43	40	42	36	36
3	AMA_BeAn70563	59	59	—	45	45	44	27	17	46	46	42	42	14	14	25	26	27	25	26	n/a	n/a	37	25	26	26	25	26	26	25	25	25	25	37	42	42	42	43	27	29	49	44	44
4	BCN_A0060209	58	58	50	—	1	1	43	46	18	17	35	35	44	44	46	46	46	46	46	n/a	n/a	42	45	45	45	45	45	45	45	45	45	45	43	35	36	36	36	45	46	25	18	17
5	BCN_98470029	57	58	50	2	—	0	43	46	17	17	35	35	44	44	46	46	46	46	46	n/a	n/a	42	45	45	45	45	45	45	45	45	45	45	43	35	36	35	36	44	45	25	18	17
6	BCN_A0070039	58	58	50	2	2	—	43	46	17	17	34	34	44	44	45	45	46	45	45	n/a	n/a	42	45	45	45	45	45	45	45	45	45	45	43	34	36	35	36	44	45	24	18	17
7	CHA_810419	53	53	44	46	47	47	—	28	43	43	41	41	27	27	29	29	30	29	29	n/a	n/a	38	28	28	28	28	28	28	28	28	28	28	38	41	41	41	41	15	30	47	43	43
8	CPX_BeAn119303	58	58	30	45	45	45	42	—	46	46	44	44	17	16	25	26	27	26	26	n/a	n/a	35	25	24	25	25	25	25	25	25	25	25	36	43	44	44	44	28	30	49	46	45
9	CTN_AVA0400135	55	55	52	34	34	34	45	47	—	2	35	35	44	44	45	46	46	46	46	n/a	n/a	42	45	45	45	45	45	45	45	45	45	45	44	38	36	36	37	44	46	22	13	13
10	CTN_AVA0400212	55	55	53	34	34	34	46	48	5	—	35	35	44	44	45	46	46	46	46	n/a	n/a	43	45	45	45	45	45	45	45	45	45	45	44	38	36	36	37	44	46	22	13	13
11	FLE_Pinheiro	17	15	57	56	56	56	52	56	56	57	—	0	41	41	42	41	42	41	41	n/a	n/a	43	42	43	42	42	42	43	42	42	42	42	42	21	30	30	32	43	43	41	37	37
12	FLE_BeAn293022	17	15	57	56	56	56	52	56	56	57	0	—	41	41	42	41	42	41	41	n/a	n/a	43	42	43	42	42	42	43	42	42	42	42	42	21	30	30	32	43	43	41	37	37
13	GTO_CVH960101	58	58	29	47	48	48	41	30	51	51	55	55	—	0	24	25	26	25	25	n/a	n/a	36	23	23	23	23	23	23	23	22	23	23	37	41	42	42	41	29	28	48	44	44
14	GTO_INH95551	57	57	29	47	48	48	41	29	50	50	55	55	1	—	24	25	26	25	25	n/a	n/a	36	23	23	23	23	23	23	23	22	23	23	37	41	42	42	41	29	28	48	44	44
15	JUN_Rumero	59	58	43	47	48	48	42	45	49	49	56	56	42	42	—	1	4	3	1	n/a	n/a	38	12	12	12	11	12	12	12	11	11	12	39	42	44	44	43	29	20	48	44	44
16	JUN_Candid1	59	59	44	47	48	49	43	46	49	49	57	57	42	43	2	—	4	3	0	n/a	n/a	39	12	12	12	11	12	12	12	11	11	12	39	42	43	43	43	29	20	48	44	44
17	JUN_MC2	59	58	43	47	48	48	42	45	49	49	56	56	42	43	1	2	—	3	4	n/a	n/a	39	13	14	14	13	13	14	13	13	13	13	40	42	45	44	43	30	21	49	46	46
18	JUN_cbaIV4454	59	58	44	47	48	48	42	46	49	49	56	56	42	43	1	2	2	—	3	n/a	n/a	38	12	12	12	11	12	12	12	11	11	12	38	41	44	44	43	29	20	48	44	44
19	JUN_XJ13	59	58	43	47	48	48	42	45	48	49	56	56	42	42	1	1	1	2	—	n/a	n/a	39	12	12	12	11	12	12	12	11	11	12	39	42	43	43	43	29	20	48	44	44
20	JUN_XJ	59	58	43	47	48	48	42	45	49	49	56	56	42	43	1	2	0	2	1	—	n/a	n/a	n/a	n/a	n/a	n/a	n/a	n/a	n/a	n/a	n/a	n/a	n/a	n/a	n/a	n/a	n/a	n/a	n/a	n/a	n/a	n/a
21	JUN_XJ44	59	58	43	47	48	48	42	45	49	49	56	56	42	43	1	2	0	2	1	0	—	n/a	n/a	n/a	n/a	n/a	n/a	n/a	n/a	n/a	n/a	n/a	n/a	n/a	n/a	n/a	n/a	n/a	n/a	n/a	n/a	n/a
22	LAT_MARU10924	47	47	54	53	53	53	49	50	54	55	45	45	50	50	54	54	54	53	54	54	54	—	37	37	37	37	37	37	37	37	37	37	19	41	42	42	40	37	39	45	42	41
23	MAC_9530537	59	59	43	48	48	48	43	45	49	50	58	58	43	43	25	26	26	25	25	26	26	52	—	2	2	2	3	2	1	2	2	1	37	42	43	44	43	28	19	48	44	43
24	MAC_Mallele	58	58	44	49	49	49	43	45	48	49	56	56	43	43	26	27	26	26	26	26	26	52	5	—	2	1	2	2	1	2	1	1	38	43	43	44	43	28	19	48	43	43
25	MAC_Chicava	58	58	44	48	48	48	43	45	49	49	56	56	44	44	26	27	26	26	26	26	26	52	5	4	—	2	3	0	1	2	2	1	37	43	43	44	43	28	19	48	44	44
26	MAC_9430084	58	58	43	48	48	48	43	45	49	50	56	56	43	44	26	27	26	25	26	26	26	52	3	3	3	—	1	1	1	1	1	1	38	43	43	44	43	27	19	48	44	44
27	MAC_200002427	58	58	44	48	48	48	44	45	48	49	56	56	43	44	26	27	26	26	26	26	26	52	5	4	4	3	—	2	2	1	1	2	38	43	43	44	43	28	20	48	44	44
28	MAC_9301012	58	58	44	48	48	48	43	46	49	49	56	56	44	44	26	27	26	26	26	26	26	53	5	4	1	3	4	—	1	2	2	1	37	43	43	44	43	28	19	49	44	44
29	MAC_MARU216606	58	58	43	48	48	48	43	45	49	49	57	57	43	44	26	27	26	26	26	26	26	52	4	4	1	2	4	2	—	1	1	0	38	43	43	44	43	28	19	48	44	44
30	MAC_MARU222688	57	57	44	49	49	49	43	45	49	50	56	56	43	43	25	26	26	25	25	26	26	52	4	4	2	2	4	2	1	—	1	1	38	43	43	43	43	28	19	48	44	43
31	MAC_MARU249121	58	58	43	48	48	48	43	45	48	49	57	57	43	43	26	27	26	25	26	26	26	52	3	3	3	0	3	3	2	2	—	1	37	43	43	44	43	28	19	48	44	43
32	MAC_Carvallo	58	58	43	48	48	48	43	45	49	49	57	57	43	44	26	27	26	26	26	26	26	52	4	4	1	2	4	2	0	1	2	—	38	43	43	44	43	28	19	48	44	44
33	OLI_3229-1	47	48	55	53	53	53	49	51	53	53	47	47	50	49	52	53	53	52	52	53	53	19	51	51	51	51	51	51	51	51	51	51	—	42	42	43	40	38	39	45	43	43
34	PAR_12056	17	16	58	59	59	59	53	57	56	57	17	17	55	55	58	58	58	58	58	58	58	48	58	57	57	57	57	57	57	57	57	57	48	—	32	32	32	43	42	41	39	38
35	PIC_Munchique	17	17	58	57	57	57	55	55	57	58	20	20	57	56	58	58	58	58	58	58	58	47	58	57	58	58	57	57	58	57	58	58	47	18	—	3	28	42	43	39	35	35
36	PIC	19	19	58	58	58	58	57	56	58	58	22	22	58	57	59	60	59	59	59	59	59	48	59	58	58	58	58	58	58	58	59	58	49	19	5	—	29	41	42	39	36	36
37	PIR_VAV488	17	17	59	58	57	57	55	56	57	57	22	22	56	55	57	58	57	57	57	57	57	46	58	57	58	58	57	57	58	57	58	58	48	20	17	19	—	43	42	41	37	36
38	SAB_SPH114202	54	55	46	46	46	46	22	42	46	47	54	54	43	43	44	45	44	45	44	44	44	50	43	43	43	43	44	44	43	43	43	43	50	56	57	58	57	—	32	47	44	43
39	TAC	57	57	46	49	49	49	44	45	49	50	55	55	45	46	31	31	31	31	31	31	31	53	32	32	33	32	32	32	33	32	32	33	53	57	57	58	57	47	—	49	45	45
40	TAM_W10777	57	56	49	34	33	33	45	46	34	35	55	55	49	49	47	48	47	47	48	47	47	53	49	47	48	47	47	48	48	47	47	48	51	56	56	57	56	47	50	—	23	22
41	WWA_AV9310135	56	56	54	39	39	38	49	49	31	32	57	57	51	51	51	52	51	52	51	51	51	55	50	49	49	49	49	49	49	49	49	49	53	57	58	58	57	48	52	37	—	1
42	WWA_9310141	56	56	54	38	39	38	49	49	31	31	57	57	51	51	51	51	51	51	51	51	51	55	50	49	49	49	49	49	49	49	49	49	53	57	58	58	57	48	52	37	0	—

*Upper-right and lower-left of diagonal corresponds to uncorrected pairwise p distances for the complete nucleocapsid and complete glycoprotein amino acid sequences, respectively

‡Complete amino acid sequence is not available

Footnote for Supplemental Table

Genbank accession numbers for L amino acid sequences include: ALL (NC_010249), AMA (NC_010251), BCN (AY216503, NC_010255), CHA (EU260464), CPX (NC_010252), GTO (NC_005082), JUN (AY216507, AY619640, AY819707, DQ489718, NC_005080), MAC (AY619644, AY624354, NC_005079), OLI (NC_010250), PIC (NC_006439), PIR (NC_005897), SAB (NC_006313), TAC (NC_004292), TAM (AY924393), and WWA (AY924395).

Genbank accession numbers for Z amino acid sequences include: ALL (NC_010249), AMA (NC_010251), BCN (AY216503, NC_010255), CHA (EU260464), CPX (NC_010252), GTO (AY562388, AY562389, AY562390, AY562391, AY562392, AY562393, AY586551, AY586552, AY586553, AY586554, AY586555, NC_005082), JUN (AY216507, AY619640, AY819707, DQ489718, NC_005080), LAT (AY960333), MAC (AY619644, AY624354, AY960326, AY960327, AY960328, AY960329, AY960330, AY960331, AY960332, NC_005079), OLI (NC_010250), PIC (NC_006439), PIR (AY562384, AY562385, AY562386, AY562387, NC_005897), SAB (NC_006313), TAC (NC_004292), TAM (AY924393), and WWA (AY924395).

Genbank accession numbers for GPC amino acid sequences include: ALL (AY012686, NC_010253), AMA (NC_010247), BCN (AF512833, AY924392, NC_010256), CHA (EU260463), CPX (NC_010254), CTN (DQ865244, DQ865245), FLE (AF485257, AF512831), GTO (AY497548, NC_005077), JUN (AY619641, AY746353, D10072, DQ272266, NC_005081, U70799, U70800), LAT (AF512830), MAC (AY571959, AY619645, AY924202, AY924203, AY924204, AY924205, AY924206, AY924207, AY924208, NC_005078), OLI (NC_010248), PAR (AF485261), PIC (AF0181555, NC_006447), PIR (NC_005894), SAB (NC_006317), TAC (NC_004293), TAM (AF485263), and WWA (AF228063, AF485264).

Genbank accession numbers for NP amino acid sequences include: ALL (AY012686, NC_010253), AMA (NC_010247), BCN (AF512833, AY924392, NC_010256), CHA

(EU260463), CPX (NC_010254), CTN (DQ865244, DQ865245), FLE (AF485257, AF512831), GTO (AY497548, NC_005077), JUN (AY619641, AY746353, D10072, DQ272266, NC_005081), LAT (AF512830), MAC (AY571959, AY619645, AY924202, AY924203, AY924204, AY924205, AY924206, AY924207, AY924208, NC_005078), OLI (NC_010248), PAR (AF485261), PIC (AF0181555, NC_006447), PIR (NC_005894), SAB (NC_006317), TAC (NC_004293), TAM (AF485263), and WWA (AF228063, AF485264).
